# A Multisite Electronic Health Record Integrated Remote Monitoring Intervention for Hypertension Improvement: Protocol for a Randomized Pragmatic Comparative Effectiveness Trial

**DOI:** 10.2196/45915

**Published:** 2023-10-30

**Authors:** David R Lee, Matthew Chenoweth, Linh H Chuong, Chad W Villaflores, Miguel Cuevas, Sitaram Vangala, Jeff Borenstein, Hannah Kwak, Chidinma Chima-Melton, Maria Han, Samuel A Skootsky, Therese Chan Tack, Linda Branagan, Heather Martin, Reshma Gupta, Linda Phan, Michael A Sanchez, Mina M Malaak, Anna Dermenchyan, Kandyce N Pearson, Marine Altunyan, Peter F Barakat, Ray Pablo, Catherine Sarkisian

**Affiliations:** 1 Division of Geriatrics University of California, Los Angeles Los Angeles, CA United States; 2 Fielding School of Public Health University of California, Los Angeles Los Angeles, CA United States; 3 Department of Medicine University of California, Los Angeles Los Angeles, CA United States; 4 Division of Pulmonary and Critical Care Medicine University of California, Los Angeles Los Angeles, CA United States; 5 University of California Health Oakland, CA United States; 6 Division of General Internal Medicine University of California, San Francisco San Francisco, CA United States; 7 Telehealth Resource Center University of California, San Francisco San Francisco, CA United States; 8 Department of Pharmacy University of California Davis Health Davis, CA United States; 9 Department of Internal Medicine University of California, Davis Health Sacramento, CA United States; 10 University of California Office of the President Oakland, CA United States; 11 Ambulatory and Community Practices University of California, Los Angeles Los Angeles, CA United States; 12 University of California Health Data Warehouse University of California Health Irvine, CA United States; 13 Geriatrics Research Education and Clinical Center VA Greater Los Angeles Healthcare System Los Angeles, CA United States

**Keywords:** blood pressure, EHR, electronic health record, hypertension, hypertensive, improvement science, monitor, monitoring, randomized controlled trial, RCT, remote blood pressure monitoring

## Abstract

**Background:**

Hypertension is a major contributor to various adverse health outcomes. Although previous studies have shown the benefits of home blood pressure (BP) monitoring over office-based measurements, there is limited evidence comparing the effectiveness of whether a BP monitor integrated into the electronic health record is superior to a nonintegrated BP monitor.

**Objective:**

In this paper, we describe the protocol for a pragmatic multisite implementation of a quality improvement initiative directly comparing integrated to nonintegrated BP monitors for hypertension improvement.

**Methods:**

We will conduct a randomized, comparative effectiveness trial at 3 large academic health centers across California. The 3 sites will enroll a total of 660 participants (approximately n=220 per site), with 330 in the integrated BP monitor arm and 330 in the nonintegrated BP control arm. The primary outcome of this study will be the absolute difference in systolic BP in mm Hg from enrollment to 6 months. Secondary outcome measures include binary measures of hypertension (controlled vs uncontrolled), hypertension-related health complications, hospitalizations, and death. The list of possible participants will be generated from a central data warehouse. Randomization will occur after enrollment in the study. Participants will use their assigned BP monitor and join site-specific hypertension interventions. Cross-site learning will occur at regular all-site meetings facilitated by the University of California, Los Angeles Value-Based Care Research Consortium. A pre- and poststudy questionnaire will be conducted to further evaluate participants’ perspectives regarding their BP monitor. Linear mixed effects models will be used to compare the primary outcome measure between study arms. Mixed effects logistic regression models will be used to compare secondary outcome measures between study arms.

**Results:**

The study will start enrolling participants in the second quarter of 2023 and will be completed by the first half of 2024. Results will be published by the end of 2024.

**Conclusions:**

This pragmatic trial will contribute to the growing field of chronic care management using remote monitoring by answering whether a hypertension intervention coupled with an electronic health record integrated home BP monitor improves patients’ hypertension better than a hypertension intervention with a nonintegrated BP monitor. The outcomes of this study may help health system decision makers determine whether to invest in integrated BP monitors for vulnerable patient populations.

**Trial Registration:**

ClinicalTrials.gov NCT05390502; clinicaltrials.gov/study/NCT05390502

**International Registered Report Identifier (IRRID):**

PRR1-10.2196/45915

## Introduction

Hypertension is a marked modifiable risk factor for many adverse health outcomes, with the highest attributable risk for cardiovascular disease and death [1]. About half of the US adult population has hypertension, with the majority having uncontrolled hypertension [2]. The inadequate treatment of hypertension for certain groups is a large source of health disparities [3]. Clinically, the diagnosis of hypertension is made using office-based blood pressure (BP) measurements at several week intervals [4] and confirmed with an out-of-office reading or using ambulatory BP monitoring [5,6], as there is growing evidence showing differences in BP measurements in the office compared to the home setting [7,8]. Variations in adherence to clinical guidelines on how to properly perform BP readings, patient anxiety, environmental factors, and “white-coat hypertension” all contribute to these differences [6,9]. Additionally, many studies have shown the superiority of home BP monitoring compared to office BP monitoring in terms of cardiovascular and renal outcomes, all-cause mortality [1,8,10,11], and patient empowerment and adherence to medical therapies for hypertension [12].

Although the benefits of home BP monitoring compared to office-based monitoring are well studied, optimal methods for obtaining home BP readings are less clear. A previous study showed improvements to systolic BP at 1 year after a self-monitoring and digital intervention involving a health care provider-managed lifestyle modification and medication titration approach compared to usual care [7]. Whereas a single-center study found no difference in BP change using an electronic health record (EHR)–connected smartphone device to record BP using a standard self-monitoring strategy [13]. This randomized, comparative effectiveness trial adds to the existing literature by including multiple sites and comparing an EHR-integrated monitor with automated BP data uploads to a standard self-monitoring approach.

In developing the protocol for this study, we used the eHealth Enhanced Chronic Care Model (eCCM) [14] to shape our theoretical understanding that remote BP monitoring can improve patients’ and health care providers’ “wisdom” [15,16] to provide interventions capable of improving hypertension control. This project aligns with a joint scientific advisory on hypertension control from the American Heart Association, the American College of Cardiology, and the Centers for Disease Control and Prevention, which argued for systems-level, population-health-level initiatives to control hypertension, involving the community around a patient, leveraging data from EHRs, and patient-provider engagement [17].

This manuscript was developed to describe the protocol for this pragmatic multisite study that evaluates whether an integrated BP monitor performs better at controlling hypertension than a self-reporting strategy. We define the roles and responsibilities of key partners, study design, key outcomes, and statistical analyses.

## Methods

### Setting and Study Design

**Figure 1:**
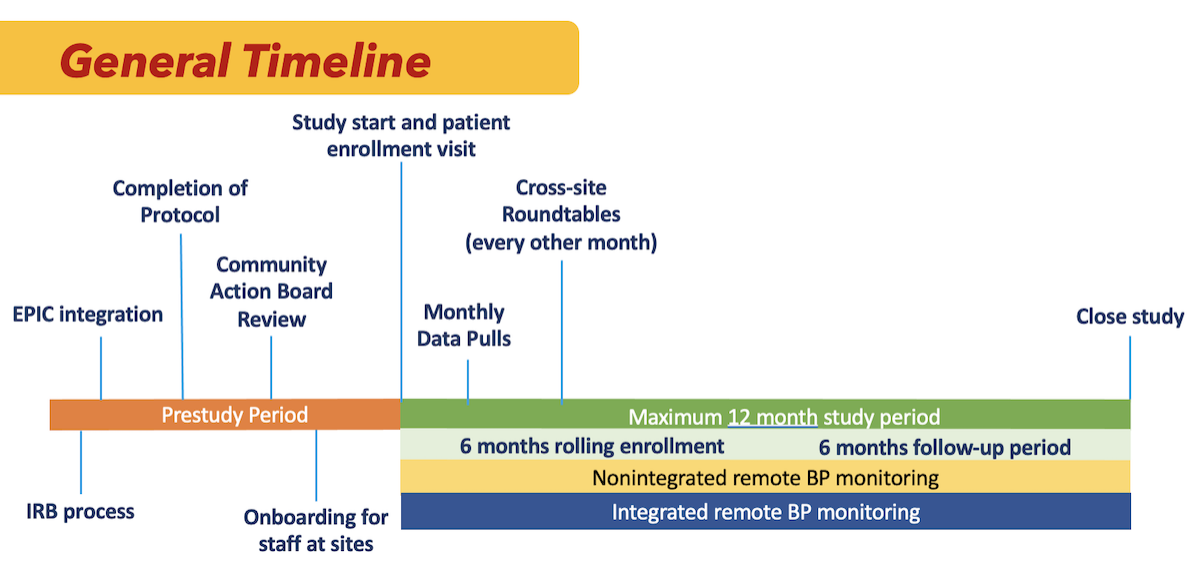
Timeline for the study. Participants will be followed for 6 months after enrollment date. BP: blood pressure; IRB: institutional review board.

The University of California (UC) Health System is a large academic health system serving a diverse patient population throughout California, the United States, and internationally. It includes teaching hospitals, affiliations with community health clinics, health professional schools, research centers, and other health care entities. This study protocol describes a randomized, comparative effectiveness trial that will be conducted at 3 of the UC Health System sites (UC Davis [UCD], UC Los Angeles [UCLA], and UC San Francisco [UCSF]). Figure 1 describes the general timeline. Enrollment will occur over 6 months after study initiation, and each participant will be followed for 6 months after enrollment.

### Defining Key Partners and Study Preparation

The UCLA Value-Based Care Research Consortium (VBCRC) will lead the planning, implementation, and evaluation of this study in collaboration with the UCLA Department of Medicine Statistics Core, the UC Office of the President (UCOP), and each UC site. The UCLA VBCRC is a team that aims to improve value in health care through partnerships with health systems and researchers. The UCOP team will provide input into study design and feasibility, as well as funding for the study, including the BP monitors. Each UC site will have a designated site champion, program manager, and IT lead (Multimedia Appendix 1 details the responsibilities of these roles). The UCLA VBCRC team will facilitate regular meetings with representatives from each team, focusing on cross-site learning, troubleshooting implementation issues, and providing updates on progress. Further evaluation of cross-site variations, barriers to implementation, and understanding of patient experiences will be obtained from qualitatively coding and analyzing these regularly scheduled meetings with the sites.

In preparation for the study, the UCLA VBCRC met with a community action board consisting of representatives from community organizations serving large numbers of diverse members with hypertension to understand patient concerns and perspectives regarding this study. Overall, the community action board supported the need for this project but raised concerns regarding proper training for using the BP monitors for participants in the study. They also reiterated the importance of providing instructional materials at various education and literacy levels, as well as individualized support for participants when needed. This feedback was provided to the sites and the BP monitor company.

### Site-Specific Hypertension Interventions

In addition to following core protocol instructions, each site will design and implement a hypertension intervention for both arms of the study. The study sites are given the autonomy to create their own site-specific hypertension intervention to fit their patient population needs and available resources. Table 1 compares interventions across each UC site, including whether the intervention is web-based only or hybrid (component of in-person and web-based), the main health care providers involved in the intervention, specific details about the intervention, discharge criteria from the program, and site-specific inclusions and exclusions.

**Table 1:** Comparison of site interventions.

	University of California, Davis	University of California, Los Angeles	University of California, San Francisco
Web-based only, hybrid (in-person and web-based)	Hybrid	Web-based only	Hybrid
BP^a^ monitor distribution plan (eg, mail, in person, etc)	Mailed directly to the patient	Mailed directly to the patient	Mailed directly to the patient
Main providers involved in the intervention	Pharmacist	Pharmacist Patient service representative Primary care provider Program supervising physician	Pharmacist Primary care provider Health care navigator
General structure of study arms	Intervention: EHR^b^ integrated remote BP monitor with pharmacist follow up Control: nonintegrated BP monitor with pharmacist follow up	Intervention: EHR integrated remote BP monitor + web-based patient portal Control: nonintegrated BP monitor + web-based patient portal	Intervention: EHR integrated remote BP monitor + usual care and follow-up Control: nonintegrated BP monitor + usual care and follow-up
Frequency of BP checks	There is no minimum per month. Ideally, at least 16 per month.	There is no minimum per month. Ideally, at least 16 per month.	There is no minimum per month; however, patients will receive daily reminders. Ideally, at least 16 per month.
Site-specific intervention	Initial visit (60 minutes): (1) The pharmacist will provide disease management (ie, monitor BP readings and help titrate medications accordingly). (2) Notify the PCP^c^ of patient participation or access to monitor. Clinic visits (1 minimum): (1) Before the pharmacist’s intervention. (2) As needed to facilitate care plans and achieve BP goals	Initial visit (40 minutes): (1) Review: PMH^d^ relevant to treatment, calculate 10-year ASCVD^e^ risk, labs, current BP management, diet and exercise, medication adherence, access to a home BP monitor, and BP measurement techniques. (2) Treatment: develop an individualized treatment plan, including starting antihypertensive medications, hypertension education, lifestyle management, and reviewing BP measurement techniques. (3) Communication: send a visit summary to PCP. (4) Visits performed by pharmacists or supervising program physicians. (5) Schedule: next visit in 2-4 weeks (monthly follow-up). Follow-up visit (20 minutes): review the individualized treatment plan, home BP measurements, and access to medications (if any).	Initial visit (40 minutes): (1) Review: PMH relevant to treatment, current blood pressure management, diet and exercise, labs, medication adherence, access to a home BP monitor, and BP measurement techniques. (2) Treatment: create an individualized plan that may include starting a new antihypertensive, dose adjustment, or tapering medications contributing to high BP, and set or reinforce target BP goals. (3) Schedule: next visit in 4 weeks. (4) Visits performed by a pharmacist, primary care doctor, nurse practitioner, or registered nurse. Follow up: every 1-2 months.
Control group	Self-report BP. Intervention is the same as above.	Self-report BP. Intervention is the same as above.	Self-report BP. Intervention is the same as above.
Discharge from the program	BP at goal for 2 consecutive visits Resistant hypertension criteria Clinical concerns develop, for example, recurrent orthostatic symptoms with systolic BP <100 mm Hg, persistent changes in labs (electrolytes and GFR^f^), significant excursions in BP after confirming measurement technique, and unstable symptoms BP is above goal after 6 months The patient requests discharge	BP at goal for 2 consecutive visits Resistant hypertension criteria Clinical concerns develop, for example, recurrent orthostatic symptoms with systolic BP <100 mm Hg, persistent changes in labs (electrolytes and GFR), significant excursions in BP after confirming measurement technique, unstable symptoms BP is above goal after 6 months The patient requests discharge	BP is well controlled. Patient request or lost to follow-up. Complicating comorbid conditions develop
Site-specific inclusion criteria	None	The participant must be active on the web-based patient portal.	The participant must be active on the web-based patient portal.
Site-specific exclusion criteria (if any)	None	None	None

^a^BP: blood pressure.

^b^EHR: electronic health records.

^c^PCP: primary care physician.

^d^PMH: patient medical history.

^e^ASCVD: atherosclerotic cardiovascular disease.

^f^GFR: glomerular filtration rate.

### Eligibility, Inclusion, and Exclusion Criteria

A complete list of eligibility, inclusion, and exclusion criteria is listed in Textbox 1. Eligible participants must be 18 years of age or older, willing and functionally able to use the BP monitor, and have access to the web-based health care patient portal (with help from another person if needed). Participants will be included if they have had an outpatient visit within the last year and 2 readings of systolic BP ≥140 mm Hg or diastolic BP ≥90 mm Hg within 6 months of each other, have a primary care physician within the health care system, and are taking three or fewer antihypertensive medications. There is no language preference or maximum age cutoff for this study.

Participants will be excluded if they had a recent BP measurement greater than or equal to 180/110 mm Hg (office BP) or greater than or equal to 175/105 mm Hg (self-monitored BP), have a history of conditions that could lead to refractory hypertension (Textbox 1), are currently incarcerated, are enrolled in home health, hospice, or a hypertension management program, have cognitive impairment that prevents them from participating in intervention activities, or have a white coat hypertension diagnosis. Individuals with BP measurements greater than or equal to 180/110 mm Hg (office BP) or greater than or equal to 175/105 mm Hg (self-monitored BP) will be excluded because they may have treatment-resistant hypertension or may need more intensive intervention than is being provided with this program.

**Complete list of eligibility, inclusion, and exclusion criteria.**

**Eligibility criteria**

- Aged 18 years and older.
- Must be willing and functionally able (with help from another person if needed) to perform remote blood pressure (BP) monitoring.
- Have access to the web-based health care portal (with help from another person if needed).

**Inclusion criteria**

- Have outpatient visit within the last 12 months, and have a previous visit within 6 months of inclusion outpatient visit with diagnosis of hypertension, defined as having 2 readings of systolic BP >140 mm Hg or diastolic BP >90 mm Hg
- Has at least one visit with primary care physician in prior year.
- Takes 0 or 3 antihypertensive prescriptions (can include pills with 2 different drugs so could be on 2 medications).

**Exclusion criteria**

- BP > 180/110 mm Hg (office) or > 175/105 mm Hg (self-monitored BP measurements)
- Pheochromocytoma
- Uncontrolled hypothyroidism or hyperthyroidism
- Renal artery stenosis
- Conn’s syndrome
- End Stage Renal Disease (ESRD)
- Chronic Kidney Disease (CKD) Stage 3b (CrCL <45) and worse
- Transplant patients—used the code that if they ever had a transplant
- Pregnancy
- Severe aortic stenosis
- Hospice or end-of-life or palliative Care
- Left Ventricular Ejection Fraction (LVEF) <30%
- Acute cardiac event in the last 3 months (eg, myocardial infarction)
- Heart block and arrhythmias
- Recurrent or symptomatic hypotension (systolic BP <100 mm Hg or diastolic BP <60 mm Hg)
- Drug and alcohol abuse
- Orthostatic hypotension (drop in systolic BP >20 mm Hg)
- Other secondary causes of hypertension
- Receiving hypertension management from other services (home health, hospice, already enrolled in hypertension management program)
- White coat hypertension

### Enrollment, Randomization, and Study Initiation

**Figure 2:**
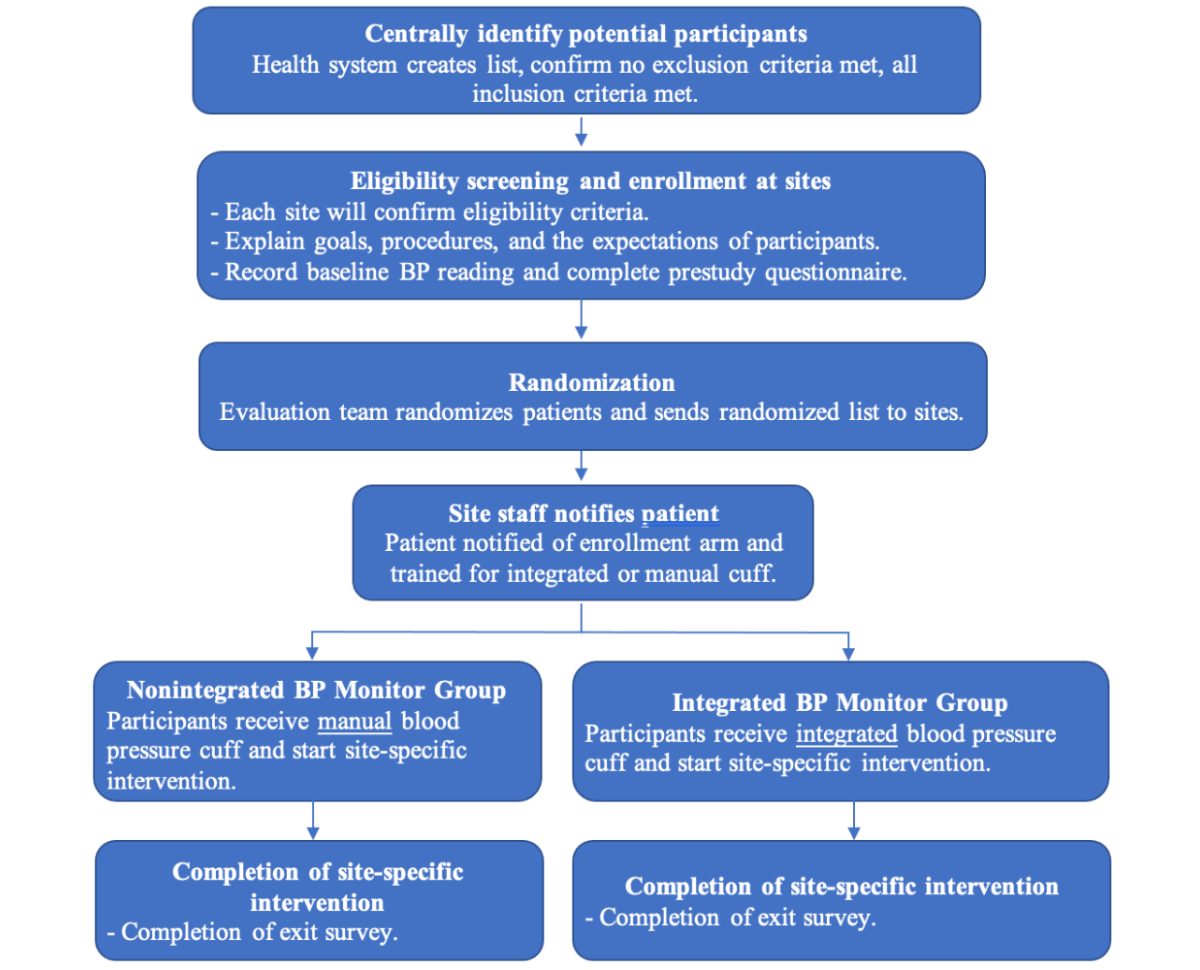
Study phases. BP: blood pressure.

Figure 2 describes the study phases, starting with the creation of the potential participant list obtained from the Center for Data-Driven Insights and Innovation (CDi2) and the UC Health Data Warehouse (UCHDW), verification of eligibility criteria, and study enrollment, which we aim to start in the second quarter of 2023. This study plans to enroll 660 participants (about 220 per site and 330 participants in each arm of the study). These power calculation estimates are based on previous literature reviews [18,19] to achieve an 80% power to detect a difference of 0.23 SDs and an anticipated 10% attrition rate. An enrollment script will be provided to each site (Multimedia Appendix 2). Randomization will occur after enrollment using a randomization allocation template within REDCap (Research Electronic Data Capture), a secure web-based platform designed to help capture data and operationalize research studies [20,21].

After enrollment and randomization, participants will receive their BP monitor (integrated or nonintegrated) and complete a prestudy questionnaire (Multimedia Appendix 3). Each UC site will then administer their site-specific hypertension intervention, which will be administered to both arms of the study. At the end of the 6-month follow-up period, a similar poststudy questionnaire evaluating participant experiences will be completed (Multimedia Appendix 4). Given the nature of the different BP management interventions, site representatives and participants will not be blinded to study arm assignments; however, the UCLA VBCRC evaluation team will be blinded.

### Blood Pressure Monitors, Electronic Health Record Integration, and Operations

Both integrated and nonintegrated BP monitors Omron 5 Series Model, BP7250 (Omron Healthcare Inc) will be directly mailed to participants’ homes, and participants will be asked to follow standard instructions for measuring BP.

EHR integration refers to the secure transfer of data from the remote BP monitor to the health system’s electronic medical record through DataHub (Omron). The process of integration will occur in collaboration with site IT leads, Omron representatives, and the Redox (Redox Inc) team. Redox is a backend integration platform that provides a standardized method for receiving and sending data between health care EHR systems and technology vendors such as Omron Healthcare Inc. Though more time- and labor-intensive to implement, the Redox platform allows for the immediate upload of data into the EHR without the use of a mobile app. For all participants with the integrated BP monitor, BP measurements will be immediately available in the EHR for intervention.

In terms of operations, the integrated BP monitors will store measurements in the provided DataHub, which will then automatically transmit BP measurements to Redox. At the backend, these BP measurements in Redox will then be transmitted to the patients’ medical records and stored as a flowsheet item in the EHR that can be acted upon by local site-specific interventions. In contrast, the nonintegrated BP monitor will require participants to self-report BP measurements to their health care provider through the site-specific web-based patient portal. Participant and staff training on how to use the integrated BP monitor, techniques for taking BP, and further technical support in multiple languages will be conducted by Omron representatives. Omron representatives will call the participant after the monitor has been delivered and follow a standardized script that includes asking the participant to take multiple BP measurements and ensuring BP readings are properly captured in the database.

### Primary and Secondary Outcomes

The primary outcome is the difference in BP from baseline compared to 6 months between study groups (integrated vs nonintegrated BP monitor), defined as a continuous measure of BP in mm Hg. Secondary outcomes include a binary measure of controlled versus uncontrolled hypertension (where uncontrolled is defined as systolic BP >130 mm Hg or diastolic BP >80 mm Hg based on American College of Cardiology and American Heart Association Practice Guidelines [22]), hypertension-related health complications by the end of the study, including acute coronary syndrome (myocardial infarction), stroke (ischemic and hemorrhagic), decompensated heart failure, syncope, electrolyte abnormalities (hyponatremia, hypokalemia, and hyperkalemia), hospitalization, and mortality (all-cause). Hypertension-related health complications will be captured using International Classification of Diseases Ninth or Tenth Revision codes after the participants have started the study.

### Statistical Analysis

For the primary outcome, linear mixed effects models will be used to compare changes from baseline home BP to 6 months from enrollment between the integrated and nonintegrated remote monitoring arms of the study. Models will include random site effects to account for the clustering of patients within sites. Models will adjust for demographic and clinical characteristics including age, sex, race, and ethnicity, as well as social vulnerability (the census tract Social Vulnerability Index). The evaluation of each arm will be based on a Wald test of the primary model term, and estimated differential changes will be reported along with a 95% CI. For the dichotomous secondary outcomes, we will use mixed effects logistic regression models with similar specifications and report differences in terms of odds ratios. A significance level of .05 will be used, and all analyses will follow the intention-to-treat principle. For patients who do not have a BP reading at 6 months, we will use the reading closest to 6 months, with a minimum 2-month period between the baseline and final reading [23,24]. Missing data will be handled through multiple imputations using pattern-mixture models. For the exploratory pre-post analysis, we will use linear mixed effects models to evaluate changes in BP. The analysis will be performed using SAS (version 9.4; SAS Institute Inc).

### Ethical Considerations

The UCLA institutional review board (IRB) approved the study (IRB #22-000036) before study initiation on May 27, 2022, and agreed to serve as the IRB of record for UC Davis and UC San Francisco. All amendments to the protocol will be submitted for approval by the IRB. Results will be presented at clinical conferences and in the scientific literature. Based on findings from this study, similar remote BP monitoring programs may be disseminated to other UC sites not included in this study.

The UCLA VBCRC team will use Observational Medical Outcomes Partnership (OMOP) Common Data Models (CDM) in collaboration with CDi2 and the UCHDW to access BP data and secondary outcome measures from the EHR during the study period. The VBCRC team will monitor data quality at monthly intervals.

## Results

All 3 clinical sites will begin enrolling participants during the second quarter of 2023. We anticipate the study to conclude in the first half of 2024 for all 3 sites. The results of this study will be available in 2024, and we aim to publish them by the end of 2024.

## Discussion

Improving hypertension outcomes depends on timely and accurate BP measurements. This study is a pragmatic trial that focuses on clinical effectiveness, testing whether an integrated BP monitor is more effective than a nonintegrated BP monitor at BP improvement while documenting cross-site variation, barriers to implementation, and patient experiences.

One limitation to this study design is the risk of contamination across study arms; it is possible that providers taking care of patients in the integrated BP monitoring arm might escalate the level of care they provide to their patients in the nonintegrated arm, biasing the study to the null. Because the major difference between the study arms is the integration itself, we do not anticipate this to be a major limitation. Another limitation is the possibility of poor fidelity to the intervention; participants in both arms may choose to not actually take their BP, limiting the impact of the interventions. As a pragmatic trial of a quality improvement intervention, there are also limits to the types of data that can be collected; for example, we will not be collecting insurance type, primary language spoken, or telehealth use, all of which would be interesting to know whether these had a moderating effect on the intervention.

Overall, the findings from this study will inform health system decision makers in determining whether to invest resources in integrated BP monitoring for their patient populations. Additionally, it could support the development of programs to evaluate whether remote patient monitoring is a viable option, particularly for geographically dispersed populations.

## Appendix

Multimedia Appendix 1 Roles and Responsibilities.

Multimedia Appendix 2 Enrollment Invitation Script.

Multimedia Appendix 3 Pre-Survey Questions.

Multimedia Appendix 4 Post-Survey Questions.

## Abbreviations

BP: blood pressure
CDi2: Center for Data-Driven Insights and Innovation
CDM: Common Data Models
eCCM: eHealth Enhanced Chronic Care Model
EHR: electronic health record
IRB: institutional review board
OMOP: Observational Medical Outcomes Partnership
REDCap: Research Electronic Data Capture
UC: University of California
UCD: UC Davis
UCHDW: UC Health Data Warehouse
UCLA: UC Los Angeles
UCOP: UC Office of the President
UCSF: UC San Francisco
VBCRC: Value-Based Care Research Consortium

## Data Availability

The data sets generated during or analyzed during this study are available from the corresponding author on request.
