# Supplementary figures and images for: A Multisite Electronic Health Record Integrated Remote Monitoring Intervention for Hypertension Improvement: Protocol for a Randomized Pragmatic Comparative Effectiveness Trial
Source: JMIR Res Protoc. 2023 Oct 30;12:e45915. doi: 10.2196/45915 (PMC10644190; doi:10.2196/45915)

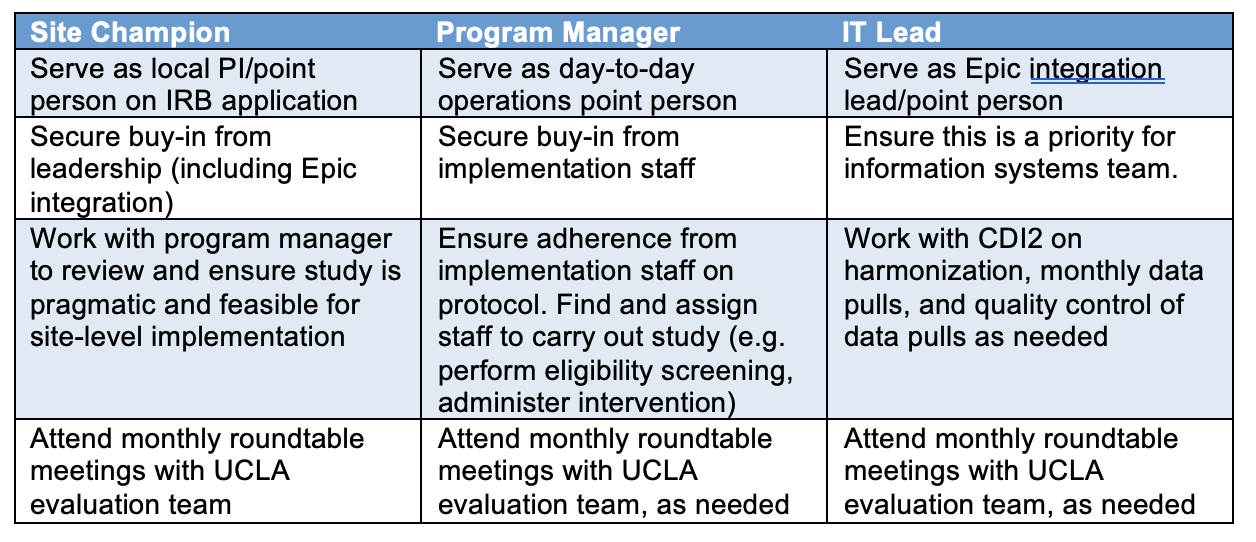

Supplement: Multimedia Appendix 1 [file resprot_v12i1e45915_app1.png]
