# Supplementary material for: A Multisite Electronic Health Record Integrated Remote Monitoring Intervention for Hypertension Improvement: Protocol for a Randomized Pragmatic Comparative Effectiveness Trial
Source: JMIR Res Protoc. 2023 Oct 30;12:e45915. doi: 10.2196/45915 (PMC10644190; doi:10.2196/45915)
Supplement: Multimedia Appendix 2 [file resprot_v12i1e45915_app2.pdf]

Enrollment Invitation Script

Record ID ______________________

UC-Wide Remote Blood Pressure Monitoring Project

Staff: Hi, my name is ____________ and I am calling to invite you to participate in a new home blood pressure monitoring program we are rolling out at multiple University of California health systems.

Can I tell you a little more about it so you can decide if you want to join?(if no, hang up. If yes, go on).

◯ Yes
◯ No

As you may have heard, checking blood pressure at home is more successful than just checking at a doctor's office. For this reason, we are trying out 2 different types of home blood pressure monitors. If you decide to participate, we will give you a new home monitor to use for six months and ask you to take your blood pressure as directed. We will also teach you how to use your blood pressure monitor and provide additional monitoring of your blood pressure, above and beyond usual care.

(insert site-specific details, for example, UCLA - virtual video appointments with pharmacists; UC Davis, provide education on how to improve your blood pressure over the course of two months , UCSF - XXX).

If you are interested, can I ask you a few questions to see if you are eligible:

◯ Yes
◯ No

1. Are you interested in participating in a home monitoring blood pressure quality improvement research project?

◯ Yes
◯ No

2. Would you be willing to participate in home blood pressure monitoring including taking your own BP at home as directed?

◯ Yes
◯ No

3. Are you currently enrolled in any other remote blood pressure monitoring program?

◯ Yes
◯ No
(Remote Blood Pressure Monitoring program that is administered by the healthcare system. It does not include patient measuring it at home.)

4. Do you have plans to move away?

◯ Yes
◯ No

5. Do you have elective surgery in next 6 months?

◯ Yes
◯ No

6. Are you willing to use either type of remote blood pressure monitor?

◯ Yes
◯ No
(Omron 7250 or Omron 9210T)

Patient is Eligible

If yes to all: Great - you meet all the criteria and are free to enroll in this project. This project has been reviewed by the UCLA Institutional Review Board and has been determined to have minimal risk. There is slight risk of you feeling anxious taking your blood pressure. There is also a chance that you would benefit from the program due to better control of your blood pressure.

Patient is NOT Eligible:

If no to any of these: I am sorry this project is not the right one for you at this time. Thank you for your time.

Do you have any questions?

If not, can I confirm: do you want to enroll in this project?

◯ Yes
◯ No

If yes, we will arrange an appointment for you to have your blood pressure taken in person (or we will do it now if this is an in-person visit) and to deliver your new home monitor to you and teach you how to use it.

If yes: great, thank you very much (scheduleBP visit)

____

If no: Sorry to hear that. In order to improve our program in the future, can you please tell me why you are turning down this opportunity? (record open-ended answer verbatim).

____

Then ask: How much would you agree or disagree with the following statement:

"I am uncomfortable with health programs that involve technology." Would you say you strongly agree, agree, disagree, or strongly disagree? (record response).

◯ Strongly Agree
◯ Agree
◯ Disagree
◯ Strongly Disagree
