# Supplementary material for: A Multisite Electronic Health Record Integrated Remote Monitoring Intervention for Hypertension Improvement: Protocol for a Randomized Pragmatic Comparative Effectiveness Trial
Source: JMIR Res Protoc. 2023 Oct 30;12:e45915. doi: 10.2196/45915 (PMC10644190; doi:10.2196/45915)
Supplement: Multimedia Appendix 3 [file resprot_v12i1e45915_app3.pdf]

Pre-Survey

Please complete the survey below.

Thank you!

Please take a few minutes to answer the following questions and return these pages to your healthcare provider during this visit. Thank you in advance for your time and we look forward to hearing from you.

Name: ______________________________

Date of Visit ______________________________

Remote Blood Pressure Monitoring Trial-Patient Survey (Pre-Intervention)

Thank you for filling out our initial questionnaire. This survey has 4 sections and a total of 23 questions. We estimate that it will take 10 minutes to complete. Please fill it out based on your own experiences, remembering that there are no right or wrong answers. Once completed, please return it to your healthcare provider before leaving the clinic today.

If you would like more information, please contact us at local contact information

Demographic Information (Source: CHIS Survey)

We would like to start by asking you some background questions

On your original birth certificate, was your sex assigned as male or female?

- ◯ Male
- ◯ Female

Are you Latino or Hispanic?

- ◯ Yes
- ◯ No

Which one or more of the following would you describe yourself as?

- ◯ White
- ◯ Black or African American
- ◯ Asian
- ◯ American Indian or Alaskan Native
- ◯ Pacific Islander
- ◯ Native Hawaiian
- ◯ Other (Specify):

Other (Specify): ______________________________

What is the highest grade of education you have completed and received credit for?

- ◯ No formal education
- ◯ Grade School
- ◯ High School or equivalent
- ◯ 4-year college or university
- ◯ Graduate or professional school
- ◯ 2-year junior or community college
- ◯ Vocational, business, or trade school

Are you now married, living with a partner in a marriage-like relationship, widowed, divorced, separated, or never married?	◯ Married<br>◯ Living with partner<br>◯ Widowed<br>◯ Divorced<br>◯ Separated<br>◯ Never married

Home Blood Pressure Monitoring

We are interested in your experiences with monitoring your blood pressure at home.

Has anyone ever shown or told you how to accurately measure you blood pressure?	◯ Yes<br>◯ No
How often are you checking your blood pressure at home? (select one)	◯ Multiple times every day<br>◯ Once every day<br>◯ Multiple times each week<br>◯ Once a week<br>◯ Once a month<br>◯ Not at all<br>◯ Other:
Other:	______________________________
What is your blood pressure goal?	◯ 120/80 or less<br>◯ 130/80 or less<br>◯ 140/90 or less<br>◯ 150/90 or less<br>◯ Other _____<br>◯ I don't know
Other:	______________________________
Are your home blood pressure readings at your goal? (select one)	◯ Mostly at my goal<br>◯ Mostly below my goal<br>◯ Mostly above my goal<br>◯ I don't know<br>◯ I don't check my blood pressure at home

Patient Efficacy

How confident are you that you can control and manage your high blood pressure? Would you say you are...	◯ Very Confident<br>◯ Somewhat Confident<br>◯ Not too confident<br>◯ Not at all confident<br>◯ Refused<br>◯ Don't Know

Technology Use Information

Are you able to use the Internet to get information from websites?	◯ Yes, at home<br>◯ Yes at another location<br>◯ Someone does this for me<br>◯ No
If you are able to get access to the internet, what is usually used to get onto the internet? (check all that apply)	☐ Computer, laptop, netbook<br>☐ Tablet (e.g. iPad)<br>☐ Cell phone<br>☐ Other (Specify):
Other:	__________________________
Have you ever used a remote monitor device to keep track of a health condition like diabetes or hypertension before?	◯ Yes<br>◯ No<br>◯ I don't know

Global Health

In general, would you say your health is:	◯ Excellent<br>◯ Very good<br>◯ Good<br>◯ Fair<br>◯ Poor
In general, would you say your quality of life is:	◯ Excellent<br>◯ Very good<br>◯ Good<br>◯ Fair<br>◯ Poor
In general, how would you rate your physical health?	◯ Excellent<br>◯ Very good<br>◯ Good<br>◯ Fair<br>◯ Poor
In general, how would you rate your mental health, including your mood and your ability to think?	◯ Excellent<br>◯ Very good<br>◯ Good<br>◯ Fair<br>◯ Poor
In general, how would you rate your satisfaction with your social activities and relationships?	◯ Excellent<br>◯ Very good<br>◯ Good<br>◯ Fair<br>◯ Poor
In general, please rate how well you carry out your usual social activities and roles. (This includes activities at home, at work and in your community, and responsibilities as a parent, child, spouse, employee, friend, etc.)	◯ Excellent<br>◯ Very good<br>◯ Good<br>◯ Fair<br>◯ Poor

To what extent are you able to carry out your everyday physical activities such as walking, climbing stairs, carrying groceries, or moving a chair?	◯ Completely<br>◯ Mostly<br>◯ Moderately<br>◯ A little<br>◯ Not at all
In the past 7 days, how often have you been bothered by emotional problems such as feeling anxious, depressed or irritable?	◯ Never<br>◯ Rarely<br>◯ Sometimes<br>◯ Often<br>◯ Always
In the past 7 days, how would you rate your fatigue on average?	◯ None<br>◯ Mild<br>◯ Moderate<br>◯ Severe<br>◯ Very severe
In the past 7 days, how would you rate your pain on average?	◯ 0. No pain<br>◯ 1.<br>◯ 2.<br>◯ 3.<br>◯ 4.<br>◯ 5.<br>◯ 6.<br>◯ 7.<br>◯ 8.<br>◯ 9.<br>◯ 10. Worst pain imaginable

Thank You!

Thank you for your time. If you have any questions or would like more information, please contact us at local contact information
