# Supplementary material for: A Multisite Electronic Health Record Integrated Remote Monitoring Intervention for Hypertension Improvement: Protocol for a Randomized Pragmatic Comparative Effectiveness Trial
Source: JMIR Res Protoc. 2023 Oct 30;12:e45915. doi: 10.2196/45915 (PMC10644190; doi:10.2196/45915)
Supplement: Multimedia Appendix 4 [file resprot_v12i1e45915_app4.pdf]

Post-Survey

Please complete the survey below.

Thank you!

Please take a few minutes to answer the following questions and return these pages to your healthcare provider during this visit. Thank you in advance for your time and we look forward to hearing from you.

Name: ________________________________

Date of Visit ________________________________

Remote Blood Pressure Monitoring Trial-Patient Survey (Post-Intervention)

Thank you for filling out our follow up questionnaire. This survey has 4 sections and a total of 19 questions. We estimate that it will take 5-10 minutes to complete. Please fill it out based on your own experiences, remembering that there are no right or wrong answers. Once completed, please return it to your healthcare provider before leaving the clinic today.

If you would like more information, please contact us at local contact information

Home Blood Pressure Monitoring

We are interested in your experiences with monitoring your blood pressure at home.

Has anyone ever shown or told you how to accurately measure you blood pressure?

- ◯ Yes
- ◯ No

How often are you checking your blood pressure at home? (select one)

- ◯ Multiple times every day
- ◯ Once every day
- ◯ Multiple times each week
- ◯ Once a week
- ◯ Once a month
- ◯ Not at all
- ◯ Other:

Other: ________________________________

What is your blood pressure goal?

- ◯ 120/80 or less
- ◯ 130/80 or less
- ◯ 140/90 or less
- ◯ 150/90 or less
- ◯ Other _____
- ◯ I don't know

Other: ________________________________

Are your home blood pressure readings at your goal? (select one)

- ◯ Mostly at my goal
- ◯ Mostly below my goal
- ◯ Mostly above my goal
- ◯ I don't know
- ◯ I don't check my blood pressure at home

Do you feel this remote blood pressure monitoring program has improved your blood pressure?

- ◯ Strongly Agree
- ◯ Agree
- ◯ Neutral
- ◯ Disagree
- ◯ Strongly Disagree

Patient Efficacy

How confident are you that you can control and manage your high blood pressure? Would you say you are...

- ◯ Very Confident
- ◯ Somewhat Confident
- ◯ Not too confident
- ◯ Not at all confident
- ◯ Refused
- ◯ Don't Know

Technology Use Information

We would like to know about how much you use information technology. This includes computers, cell phones, tablets, and any other internet connected devices.

Are you able to use the Internet to get information from websites?

- ◯ Yes, at home
- ◯ Yes at another location
- ◯ Someone does this for me
- ◯ No

If you are able to get access to the internet, what is usually used to get onto the internet? (check all that apply)

- ☐ Computer, laptop, netbook
- ☐ Tablet (e.g. iPad)
- ☐ Cell phone
- ☐ Other (Specify):

Other:

____

We are interested in understanding the burden and effort it took for you to use the blood pressure device at home.

On a scale 0-10, where 0 is the not burdensome and 10 is extremely burdensome, how would you rate how burdensome it was to measure your blood pressure at home the recommended number of times?

- ◯ 0 Not Burdensome
- ◯ 1
- ◯ 2
- ◯ 3
- ◯ 4
- ◯ 5
- ◯ 6
- ◯ 7
- ◯ 8
- ◯ 9
- ◯ 10 Extremely Burdensome

Did you require help from a caregiver or family member using the Omron device?

- ◯ Yes
- ◯ No

Global Health

In general, would you say your health is:	◯ Excellent<br>◯ Very good<br>◯ Good<br>◯ Fair<br>◯ Poor
In general, would you say your quality of life is:	◯ Excellent<br>◯ Very good<br>◯ Good<br>◯ Fair<br>◯ Poor
In general, how would you rate your physical health?	◯ Excellent<br>◯ Very good<br>◯ Good<br>◯ Fair<br>◯ Poor
In general, how would you rate your mental health, including your mood and your ability to think?	◯ Excellent<br>◯ Very good<br>◯ Good<br>◯ Fair<br>◯ Poor
In general, how would you rate your satisfaction with your social activities and relationships?	◯ Excellent<br>◯ Very good<br>◯ Good<br>◯ Fair<br>◯ Poor
In general, please rate how well you carry out your usual social activities and roles. (This includes activities at home, at work and in your community, and responsibilities as a parent, child, spouse, employee, friend, etc.)	◯ Excellent<br>◯ Very good<br>◯ Good<br>◯ Fair<br>◯ Poor
To what extent are you able to carry out your everyday physical activities such as walking, climbing stairs, carrying groceries, or moving a chair?	◯ Completely<br>◯ Mostly<br>◯ Moderately<br>◯ A little<br>◯ Not at all
In the past 7 days, how often have you been bothered by emotional problems such as feeling anxious, depressed or irritable?	◯ Never<br>◯ Rarely<br>◯ Sometimes<br>◯ Often<br>◯ Always
In the past 7 days, how would you rate your fatigue on average?	◯ None<br>◯ Mild<br>◯ Moderate<br>◯ Severe<br>◯ Very severe

In the past 7 days, how would you rate your pain on average?	◯ 0. No pain<br>◯ 1.<br>◯ 2.<br>◯ 3.<br>◯ 4.<br>◯ 5.<br>◯ 6.<br>◯ 7.<br>◯ 8.<br>◯ 9.<br>◯ 10. Worst pain imaginable
Would you recommend this operation to someone else?	◯ Definitely Yes<br>◯ Probably Yes<br>◯ Unsure<br>◯ Probably Not<br>◯ Definitely Not

Thank You!

Thank you for your time. If you have any questions or would like more information, please contact us at local contact information
